# Public health impact of a novel smoking cessation outreach program in Ontario, Canada

**DOI:** 10.1186/s12889-018-6012-6

**Published:** 2018-09-14

**Authors:** Peter Selby, Sabrina Voci, Laurie Zawertailo, Dolly Baliunas, Rosa Dragonetti, Sarwar Hussain

**Affiliations:** 1 Centre for Addiction and Mental Health, 175 College St, Toronto, ON M5T 1P7 Canada; 2 Dalla Lana School of Public Health, University of Toronto, 155 College Street, Toronto, ON M5T 3M7 Canada; 3 Department of Psychiatry, University of Toronto, 250 College Street, Toronto, ON M5T 1R8 Canada; 4 Department of Family and Community Medicine, University of Toronto, 500 University Avenue, Toronto, ON M5G 1V7 Canada; 5 Department of Pharmacology and Toxicology, University of Toronto, 1 King’s College Circle, Toronto, ON M5S 1A8 Canada

**Keywords:** Smoking, Tobacco, Smoking cessation, Nicotine replacement therapy

## Abstract

**Background:**

Provision of evidence-based smoking cessation treatment may contribute to health disparities if barriers to treatment are greater for more disadvantaged groups. We describe and evaluate the public health impact of a novel outreach program to improve access to smoking cessation treatment in Ontario, Canada.

**Methods:**

We partnered with Public Health Units (PHUs) located across the province to deliver single-session workshops providing standardized evidence-based content and 10 weeks (2007–2008) or 5 weeks (2008–2016) of nicotine replacement therapy (NRT). Participants completed a baseline assessment and were followed up by phone or e-mail at 6 months. We used the RE-AIM (Reach, Effectiveness, Adoption, Implementation and Maintenance) framework to evaluate the public health impact of the program from 2007 to 2016. Given the iterative design and changes in implementation over time, data is presented annually or bi-annually.

**Results:**

There were 26,122 enrollments from 2007 to 2016. Between 31 and 442 workshops were held annually. The annual *reach* was estimated to be 0.1–0.3% of eligible smokers in Ontario. Participants were older, smoked more heavily, had a lower household income, were more likely to be female and be diagnosed with a mood or anxiety disorder, and less likely to have a postsecondary degree compared to average Ontario smokers eligible for participation. The intervention was *effective*; at 6-month follow-up 22–33% of respondents reported abstinence from smoking. *Adoption* by PHUs was 81% by the second year of operation and remained high (72–97%) thereafter, with the exception of 2009–2010 (33–56%) when the program was temporarily unavailable to PHUs due to lack of funding. *Implementation* at the organizational level was not tracked; however, at the individual level, approximately half of participants used most or all of the NRT received. On average, *maintenance* of the program was high, with PHUs conducting workshops for 7 of the 10 years (2007–2016) and 4 of the 5 most recent years (2012–2016).

**Conclusions:**

The smoking cessation program had a high rate of adoption and maintenance, reached smokers over a large geographic area, including individuals more likely to experience disparities, and helped them make successful quit attempts. This novel model can be adopted in other jurisdictions with limited resources.

## Background

Despite the availability of smoking cessation treatments that increase long-term quit success [1, 2], a majority of quit attempts are made unassisted [3]. Low rates of treatment utilization have prompted various strategies to increase demand and reduce disparities in access to pharmacotherapy and behavioural support. For example, nicotine replacement therapy (NRT) products have been made available over-the-counter, coverage for NRT and other cessation medications has expanded [4, 5] and many quitlines provide free NRT with counselling support [6]. This has led to increased NRT use [5, 7–10] and rates of cessation [8, 11]. However, barriers to access still exist for some populations with negative social determinants of health, leading to intervention-generated inequalities [12, 13].

In 2005, the Smoking Treatment for Ontario Patients (STOP) program was established to distribute free NRT to smokers wanting to quit across the province, including rural and remote areas. For more than a decade, the STOP program has made use of and evaluated various methods for distribution of free NRT including large-scale mail-outs [14] as well as providing free NRT with brief counselling through community pharmacies [15], addiction treatment agencies [16] and primary care settings [17]. Since its inception, the program’s approach has been to use both existing healthcare infrastructure as well as new and innovative means to reach smokers from all parts of the province, especially those experiencing socioeconomic and health-related disparities such as lower income or concurrent mental illness.

In 2006, the STOP program partnered with Ontario’s Public Health Units (PHUs) to pilot a program offering individual or group counselling and NRT. PHUs are official health agencies that administer public health programs and services to the community. Every urban and rural municipality (and its surrounding area) in Ontario is served by a PHU, so they have wide reach across the province. The program provided eligible smokers in the local catchment area with up to 10 weeks of NRT and multiple counseling sessions with a PHU staff member trained in smoking cessation counselling. Though all 36 PHUs in Ontario were invited, only 12 implemented the program, as many did not have the resources or capacity to participate. At the end of the pilot program, feedback from the PHUs that participated highlighted benefits of the program including increased access to cessation treatment and increased demand for existing cessation services. However, many PHUs did not have the time or resources available to handle the additional workload on an ongoing basis. The funder was willing to provide some but not all the resources necessary for such a program. This required innovation and adaptation of the intervention to fit the setting without losing the active ingredients which were access to NRT, brief behavioural support and self-help material.

Mobile and pop-up clinics have been utilized as an innovative method to bring healthcare services to local communities to reduce or eliminate financial and other access barriers to health care among underserved communities and disadvantaged populations such as the homeless, uninsured and those living in rural or remote communities [18–21]. They have been used to deliver a wide range of health services such as vision, dental, and general medical care [19, 21]; screening for various diseases [22–27]; as well as psychiatric crisis services [28] and treatment for alcohol and drug abuse [29]. However, to our knowledge, no mobile or pop-up clinic has been designed to provide free pharmacotherapy and brief behavioural support to assist with smoking cessation. In order to provide treatment in partnership with PHUs while minimizing demands on their time and resources, in 2007 we developed a new outreach program (STOP on the Road) whereby, similar to a mobile or pop-up clinic, we travelled to communities across Ontario and partnered with the local PHU to deliver a brief group smoking cessation intervention.

The decision was supported by the absence of evidence that group cessation support is less effective than individual support and evidence that is more effective than self-help alone [30, 31]. The intervention consisted of a 1-h psychoeducational presentation to the group, covering topics such as behavioural strategies for quitting, followed by a 5- to 15-min consultation with each individual to review completed assessment forms and dispense NRT with self-help materials. Over time we assisted PHUs in building the capacity to offer the intervention without our on-site presence, but with ongoing operational and financial support.

The objective of this paper is two-fold: (1) describe the use of a theory-guided approach to the implementation of a smoking cessation outreach program and (2) evaluate its public health impact over 10 years (2007–2016) using the RE-AIM framework [32]. Recognizing that demonstrating treatment efficacy in highly controlled research settings does not necessarily translate into effectiveness in real-world settings, the RE-AIM model was developed to evaluate public health interventions based on five factors conceptualized as determinants of overall impact: reach, effectiveness, adoption, implementation and maintenance [32]. Accordingly, the model predicts public health interventions will have greater impact if they reach the target population, produce the intended outcomes in the long-term, and are easily implemented consistently and sustainably over time [32].

## Methods

### STOP on the road program design and implementation

A continuous quality improvement approach, with regular evaluation and feedback, was used to inform the iterative design of the program and its implementation. We based our activities on implementation best practices described by Fixen et al. [33]. This included the following stages: (1) exploration, (2) installation, (3) initial implementation, (4) full implementation, and (5) expansion and scale-up. We also used the three components of implementation drivers—namely competency drivers, organization and leadership supports [33]. The program was approved by the Research Ethics Board of the Centre for Addiction and Mental Health.

#### Partnering with PHUs to deliver workshops in communities across Ontario

For installation and early implementation of the intervention STOP program staff (research assistants) would travel to communities across Ontario to deliver workshops in partnership with PHUs. A combination of NRT and behavioural intervention was provided during these single-session workshops. Providing the workshop in a group setting allowed treatment to be provided to up to 50 individuals in a brief period of time, requiring less time and fewer resources than individual support. STOP and the PHU arranged a mutually convenient time and date for the workshop. The PHU was responsible for locating and booking an appropriate venue, promoting the workshop, and screening prospective workshop participants for eligibility. STOP provided PHUs with template recruitment posters and ads (on air and in print) to advertise the workshop, and PHUs provided a telephone number individuals could call for screening and registration. STOP program staff travelled to the workshop venue and were responsible for setting up and conducting the workshop, ensuring consent and baseline assessment forms were completed, delivering the psychoeducational presentation, and handing out NRT kits. The STOP program was also responsible for conducting follow-up surveys and evaluating treatment outcomes and participant satisfaction.

#### Building capacity for independent delivery of workshops with off-site support

Starting in 2011, in order to increase the number of workshops and ensure more flexibility in scheduling, we adapted the program so workshops could be conducted without STOP program staff in attendance. A PHU staff member was able to facilitate the workshop if they had completed our TEACH (Training Enhancement in Applied Cessation Counselling and Health) accredited certificate program in cessation counselling, offered by the Centre for Addiction and Mental Health and University of Toronto [34], or equivalent training in tobacco cessation treatment. To allow PHU staff to distribute NRT, a medical directive and contract signed by the PHU’s medical officer of health were required. PHUs that delivered workshops on their own also received operations training and ongoing administrative and coaching support from STOP. For PHUs unable to deliver workshops on their own, STOP staff were still available to deliver a limited number of workshops (based on resources available). Even when STOP staff led workshops, any TEACH-trained PHU staff was encouraged to facilitate the group presentation to gain experience and increase their confidence in conducting the workshops independently. Another option provided for the few PHUs that did not have a medical directive and could not dispense NRT was for PHU staff to present the workshop and for STOP to mail the NRT kits directly to participants after the workshop.

**Fig. 1:**
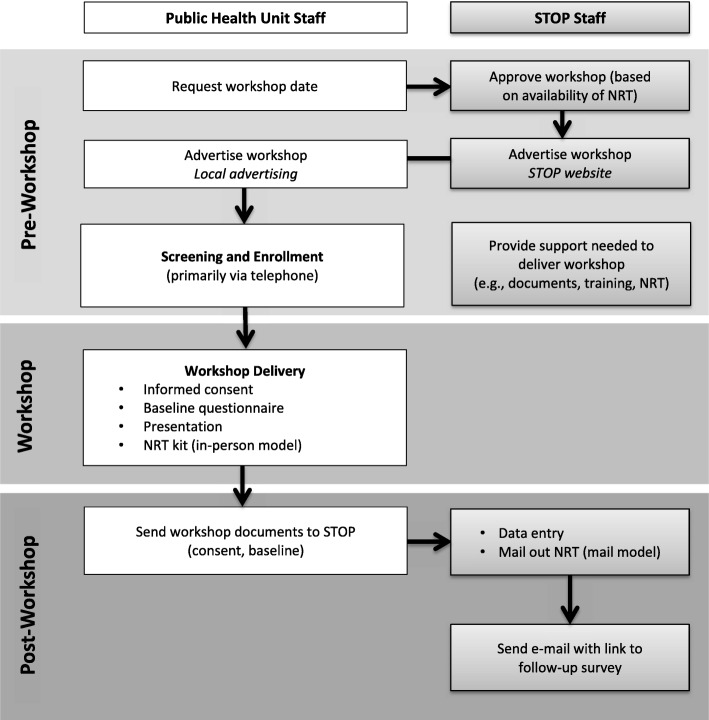
Flow diagram of workshop activities for public health and STOP staff

The STOP program continues to provide ongoing support to PHUs running workshops by providing training in program implementation as needed and working to identify the number of facilitators that are TEACH-trained who can deliver workshops in their communities. This is a necessary step given staff turnover. STOP also continues to conduct evaluations of the program and follow-up of participants to track outcomes, and regularly provides feedback to individual PHUs about their participant enrollment and outcomes. See Fig. 1 for a flowchart of activities in the current implementation model.

### Intervention

#### Smoking cessation workshop

The workshops were typically 2–3 h long. At the beginning of the workshop, participants were asked to provide written informed consent and complete a baseline assessment questionnaire. A 1-h presentation followed that covered various aspects of smoking cessation, including behavioural strategies for quitting and proper use of NRT. After the presentation, each participant had a brief 5- to 15-min consultation with STOP/PHU staff to ensure consent and assessment forms were completed, confirm eligibility, and select type of NRT from the options available. Selection of NRT was based on participant preference and previous use of NRT, along with program recommendations based on number of cigarettes smoked per day. Participants received a kit consisting of a supply of free NRT, an evidence-based self-help booklet on quitting smoking, a list of frequently asked questions about NRT, a list of additional smoking cessation resources, and STOP program contact information for questions and inquiries*.*

#### Nicotine replacement therapy

Initially, a 10-week supply of monotherapy (patch, gum, or inhaler) was provided as per manufacturers’ recommended duration of use at the time. Quit rates after providing 10 weeks of NRT were similar to those observed after providing 5 weeks of mail-out NRT in Ontario [14]. Therefore, the NRT supply was reduced to 5 weeks in 2008 to enable us to reach a greater number of smokers given limited resources. Between 2007 and 2011, a choice of nicotine patch and various short-acting forms of NRT were offered; most participants selected nicotine patch (data not shown). Since 2011, only NRT patches have been provided at workshops in either a higher dose kit (3 weeks of 21 mg, 1 week of 14 mg, and 1 week of 7 mg) or a lower dose kit (3 weeks of 14 mg and 2 weeks of 7 mg).

### Evaluation timeframe

The evaluation timeframe was January 29, 2007 (first workshop) to December 31, 2016.

### Sample

Workshop participants were required to be residents of Ontario; at least 18 years old; smoke 10 or more cigarettes per day; be willing to set a quit date within 30 days; and not have any medical contraindication to NRT (i.e., pregnant, breastfeeding, major cardiovascular incident within the past 2 weeks). Individuals were able to enroll in more than one workshop over the ten-year period, thus any reference to the number of *participants* or *individuals* should not be taken to mean unique individuals. Though this evaluation focuses on PHUs, the primary implementers of STOP on the Road workshops, participants that attended workshops through other community health providers in Ontario (e.g., Community Health Centres, Family Health Teams) are included in the sample.

### Data collection

The baseline assessment questionnaire collected data on participant demographics, smoking characteristics, and medical/psychiatric history. All participants who provided contact information were invited at 6 months after the end of treatment to complete a follow-up survey and were asked about quit attempts made and current smoking status. Follow-up was conducted either by telephone or email.

### Measures and data analysis

Given the iterative design and changes over time, data are presented annually or bi-annually. Missing data were not imputed and analyses were conducted with complete cases. Data were analyzed using SPSS version 24 [35].

#### Reach

*Reach* refers to the number and proportion of eligible individuals that are recruited to participate in an intervention and how representative they are of the target population [32]. Reach was assessed by determining the number of participants that enrolled in workshops and assessing how representative they were of the target population by comparing them with data from the representative, population-based Canadian Community Health Survey (CCHS) [36–39]. To correspond with workshop eligibility criteria, we selected a subsample from the CCHS that were 18 years or older, residing in Ontario, that smoked at least 10 cigarettes daily. Comparisons were made for each year between 2007 and 2014 on several characteristics (sex, age group, education, cigarettes/day, income, education, and incidence of mood/anxiety disorders). CCHS data for public use was only available up to 2014 at the time of this analysis.

To determine reach in rural areas, we used Rurality Index of Ontario (RIO2008) scores [40] to classify each participant’s residential location as urban or rural. Scores range from 0 (most urban) to 100 (most rural) and have been calculated for census subdivisions based on population size and density, and travel times to the nearest basic and advanced referral centres. Participants’ postal codes were linked to a census subdivision using the Statistics Canada Postal Code Conversion File [41]. Communities with a score of ≥40 were classified as rural, which is the cut-off used by the provincial government to determine eligibility for physician recruitment incentives [42]. Not all census subdivisions have been assigned a RIO score, and not all postal codes could be linked to a census subdivision; therefore, not all residential locations could be classified as urban or rural.

#### Effectiveness

*Effectiveness* is the extent to which an intervention achieves its intended outcomes [32]. The effectiveness of the intervention was assessed by examining self-reported 7-day point prevalence abstinence at 6-month follow-up, defined as not having smoked even a puff in the past 7 days. Demographic and smoking characteristics of workshop participants varied over time, and this may have had an impact on quit outcome, therefore we calculated annual quit rates standardized by gender (male or female), mental health diagnosis (yes or no), time to first cigarette (≤5 or 6+ minutes) and cigarettes/day (10–19 or 20+) to facilitate comparison of annual quit rates.

#### Adoption

Similar to the individual-level measure of reach, *adoption* assesses the number, proportion and representativeness of settings and staff willing to implement a program [32]. Adoption was assessed by examining the number and proportion of PHUs in Ontario that delivered a workshop in a particular year.

#### Implementation

At the organizational level, *implementation* refers to the extent that an intervention is consistently delivered as intended, as well as the time and costs involved, while at the individual level it refers to participant adherence [32]. With respect to implementation, fidelity to the intervention protocol was encouraged but not formally tracked, nor were time and costs shared with partnering organizations. At the individual level, we cite our previous findings regarding the level of adherence to the supply of NRT provided.

#### Maintenance

*Maintenance* at the individual level is an assessment of long-term outcomes (6 months or longer) [32] and was also, as with effectiveness, assessed by 7-day point prevalence abstinence at 6 months. At the organizational level, maintenance refers to how long an intervention program is sustained over time and the extent to which it becomes part of routine practice. Program maintenance at the organizational level was assessed by determining the number of years PHUs offered the program.

## Results

### Reach

There were a total of 26,122 enrollments in a smoking cessation workshop from January 2007 to December 2016. Of these, 98.2% (*n* = 25,369) attended a workshop through a PHU, 2.5% (*n* = 652) through another health related organization, and for 0.4% (*n* = 101) type of organization was unknown. See Table 1 for the number of enrollments each year. The number of enrollments was limited by resources available to purchase NRT and at times demand exceeded capacity; thus, with additional resources the program may have had even greater reach. Between 2009 and 2010, due to changes in political and bureaucratic processes in Ontario, a temporary funding gap led to a decrease in the number of workshops and therefore enrollments. The annual participation rate was 0.1–0.3% of daily cigarette smokers in Ontario (10+ cigarettes/day, 18 years and older), from 2007 to 2014 [36–39] (see Table 1).

**Table 1:** Reach and Adoption of Smoking Cessation Workshops in Ontario, Canada: 2007–2016

Year	Number of workshops (range for individual PHUs)	Reach		Adoption	
		Number of enrollments (range for individual PHUs)	Proportion of eligible smokers in Ontario reached^a^	Number of PHUs that partnered with STOP to deliver workshops	Number of partnering PHUs that delivered workshops without STOP on-site
2007	64 (1–7)	2927 (13–673)	0.2%	20 (56%)	N/A
2008	109 (1–7)	3082 (6–241)	0.2%	29 (81%)	N/A
2009	31 (1–6)	821 (4–153)	0.1%	12 (33%)^b^	N/A
2010	69 (1–13)	1788 (5–192)	0.2%	20 (56%)^b^	N/A
2011	85 (1–8)	2675 (28–219)	0.2%	31 (86%)	1/31 (3%)
2012	107 (1–17)	1981 (14–358)	0.2%	26 (72%)	6/26 (23%)
2013	149 (1–15)	2700 (5–300)	0.2%	35 (97%)	22/35 (63%)
2014	286 (1–25)	3583 (3–302)	0.3%	33 (92%)	29/33 (93%)
2015	252 (1–29)	2876 (4–459)	N/A	30 (83%)	28/30 (93%)
2016	442 (1–53)	3689 (5–485)	N/A	29 (81%)	29/29 (100%)
2007–2016	Total = 1594	Total = 26,122	Average = 0.2%	Total = 36/36^c^	Total = 34/36^c^

In addition to workshops held in partnership with PHUs, the total number of workshops and enrollments also includes workshops held in partnership with a small number of other healthcare-related organizations

*N/A* Not available, *PHU* Public Health Unit

^a^Based on Canadian Community Health Survey estimates of daily smokers (10+ cigarettes/day; at least 18 years old); not available for 2015–2016 at time of analysis [36–39]

^b^Fewer workshops due to funding gap for period between 2009 and 2010

^c^At least one year between 2007 and 2016

Table 2 presents baseline demographic and smoking characteristics for participants. Compared with adult daily smokers (10+ cigarettes/day) in Ontario in 2007–2014, workshop participants were older, smoked more heavily, had a lower household income, and were more likely to be female and be diagnosed with a mood or anxiety disorder. Workshop participants were also more likely to report high school diploma as the highest level of education completed and less likely to report a postsecondary degree, except in the year 2013. Note that the cutoff points for income categories in the CCHS varied very slightly ($0–$19,999; $20,000–$39,999; $40,000+) from those used by STOP (see Table 2).

**Table 2:** Smoking Cessation Workshop Participant Demographics and Smoking Characteristics: 2007–2016

	2007–2008 (*n* = 6009)	2009–2010 (*n* = 2609)	2011–2012 (*n* = 4656)	2013–2014 (*n* = 6283)	2015–2016 (*n* = 6565)
Sex, % (n)					
Female	57.9 (3477)	54.8 (1419)	54.6 (2539)	55.0 (3444)	56.2 (3675)
Male	42.1 (2526)	45.2 (1171)	45.4 (2115)	45.0 (2814)	43.8 (2859)
Age (yrs), % (n)					
18–29	8.0 (476)	8.1 (208)	8.8 (408)	8.8 (551)	7.0 (460)
30–44	29.0 (1734)	26.0 (671)	25.4 (1177)	26.2 (1635)	22.6 (1472)
45–59	46.1 (2750)	46.9 (1210)	47.6 (2207)	46.5 (2905)	46.7 (3050)
60–74	16.1 (959)	18.0 (465)	17.2 (799)	17.3 (1077)	22.3 (1457)
75+	0.9 (51)	1.0 (27)	1.0 (47)	1.2 (73)	1.3 (88)
Annual household income before tax (C$), % (n)					
≤ 20,000	28.9 (1722)	34.4 (882)	38.6 (1780)	36.4 (2276)	35.9 (2345)
20,001–40,000	20.6 (1224)	20.8 (533)	21.6 (998)	19.9 (1242)	20.6 (1349)
> 40,000	40.7 (2424)	36.1 (924)	30.2 (1395)	29.2 (1828)	28.7 (1873)
Not reported	9.8 (583)	8.7 (224)	9.6 (443)	14.5 (907)	14.8 (966)
Education, % (n)					
Less than high school diploma	22.1 (1319)	23.4 (599)	23.8 (1104)	21.1 (1296)	21.3 (1374)
High school diploma	47.8 (2847)	46.8 (1197)	46.4 (2149)	41.6 (2562)	46.5 (3006)
Post-secondary degree	30.1 (1795)	29.8 (761)	29.7 (1376)	37.3 (2297)	32.2 (2080)
Urban/rural residence, % (n)^a^					
Urban	81.2 (4420)	75.0 (1791)	82.7 (3634)	82.2 (4516)	90.9 (5293)
Rural	18.8 (1022)	25.0 (597)	17.3 (761)	17.8 (976)	9.1 (528)
Cigarettes/day, % (n)					
< 20	39.9 (2400)	36.3 (946)	37.8 (1758)	37.1 (2331)	36.8 (2409)
20+	60.1 (3609)	63.7 (1663)	62.2 (2898)	62.9 (3947)	63.2 (4143)
Time to first cigarette after waking (min), % (n)					
≤ 5	43.1 (2583)	44.9 (1160)	43.5 (2017)	43.1 (2691)	43.7 (2845)
6–30	43.6 (2617)	43.7 (1128)	46.7 (2164)	45.3 (2829)	42.7 (2784)
31+	13.3 (797)	11.4 (295)	9.8 (456)	11.6 (724)	13.6 (885)
Lifetime diagnosis of psychiatric disorder, % (n)^b^					
1+	36.7 (2203)	40.6 (1047)	46.7 (2150)	46.9 (2891)	50.7 (3183)
None	63.3 (3794)	59.4 (1532)	53.3 (2452)	53.1 (3267)	49.3 (3090)

Sample sizes vary due to missing data

^a^Based on Rurality Index for Ontario score [40]: 0–39 = urban, 40–100 = rural

^b^Self-reported diagnosis of depression, anxiety, bipolar disorder, and/or schizophrenia

### Effectiveness

See Table 3 for 6-month follow-up survey response rates and self-reported quit outcomes each year. Response rate at 6-month follow-up varied, ranging from 14 to 57%. The 7-day point prevalence abstinence rate at 6-month follow-up among survey respondents ranged from 22 to 33%. Standardized quit rates were very similar and ranged from 23 to 33%.

**Table 3:** Self-Reported Quit Outcomes at 6-Month Follow-Up (Effectiveness and Maintenance): 2007–2016

Year of workshop	Survey mode	Response rate	Crude 7-day point prevalence abstinence	Standardized 7-day point prevalence abstinence^a^
2007	Telephone	34.1% (999)	23.8% (232)	23.8%
2008	Telephone or IVR	21.8% (672)	28.2% (189)	28.1%
2009	Telephone or IVR	18.4% (151)	28.7% (43)	28.1%
2010	Telephone	56.5% (1010)	21.9% (217)	22.5%
2011	E-mail or telephone	49.7% (1330)	24.5% (325)	25.4%
2012	E-mail or telephone	50.6% (1003)	23.6% (237)	23.8%
2013	E-mail	19.4% (525)	27.7% (145)	27.3%
2014	E-mail	14.1% (505)	28.4% (142)	27.7%
2015	E-mail	14.7% (424)	35.0% (145)	33.1%
2016	E-mail	13.7% (507)	32.7% (164)	32.4%

*IVR* interactive voice response

^a^Standardized by gender, cigarettes/day, time to first cigarette upon waking and mental health diagnosis, based on 2007 sample

### Adoption

The number and proportion of PHUs that offered a workshop is presented by year in Table 1. By the second year, the majority of PHUs were partnering to offer workshops and adoption by PHUs since then has been consistently high, with the exception of 2009–2010 when, due to a funding gap, the program was temporarily unavailable (no workshops were offered by PHUs from April 19, 2009 to February 21, 2010). Within 3 years nearly all PHUs adopted one of the two new models (workshops and dispense NRT on their own or have it mailed out to participants by STOP), and within 5 years 100% of PHUs were delivering workshops without STOP staff presence onsite.

### Implementation

Though not formally assessed, an overview of some time and costs involved are described. Time needed to set-up and run a workshop was approximately 3 h. Additional time required included: (1) PHU staff: time required to find and book an appropriate venue, as well as recruit and screen participants; and (2) STOP staff: travel to workshops by car or airplane, ranging from locations within the same city to as far within the province as approximately 1500 km. STOP has consistently covered advertisement, room rental and refreshment costs (if needed) for workshops, in addition to the cost of NRT kits. STOP was previously required to also budget for travel expenses incurred, but this has been eliminated with the new implementation model. The new implementation model does require a budget for mail out of NRT kits for a small number of PHUs that cannot dispense NRT. Personnel costs for STOP included one full-time project coordinator to support the PHUs in planning the workshops and ensuring the NRT and other materials were sent to each health unit in advance of the workshop. The coordinator also updated the content of the workshop based on feedback from PHU staff, participant evaluations and recent evidence. Earlier iterations also hired research assistants to assist with workshop delivery (two STOP staff were present at each workshop) and telephone follow-up calls. The only costs PHUs invested were personnel costs for time needed for training, implementation of the workshop (including planning, participant screening and delivery) and communication with STOP staff.

Consistent delivery of the intervention was encouraged, but not monitored or assessed formally. Contents of the NRT kits were identical across all sites and they were delivered to partnering organizations already pre-assembled and sealed. In addition, presentation slides and accompanying speaker’s notes were provided in a format that could not be modified (PDF file).

At the individual level, amount of the NRT used (adherence) varied [43]. At the end of the 5- or 10-week treatment period, approximately half of those that received 10 weeks of NRT [43] or 5 weeks (unpublished data, manuscript in preparation) had used most or all of the supply provided. Various reasons were cited for using less than the full supply, most commonly side effects or relapse back to smoking [43].

### Maintenance

At the individual level, maintenance of the intervention effect is reported above as the abstinence rate at 6-month follow-up. At the organizational level, on average, PHUs offered workshops for 7 of the 10 years (2007–2016) and 4 of the 5 most recent years (2012–2016). The number of workshops per year has also increased (see Table 1). As such, workshops have been offered both relatively consistently and increasingly regularly, suggesting they have become a more routine part of practice at partnering PHUs.

## Discussion

The use of implementation science with a focus on defining the innovation and addressing stages and drivers of implementation have led to a unique scaled-up smoking cessation program in Ontario, Canada with demonstrated public health impact. The RE-AIM evaluation indicated the program had a high level of adoption that was largely maintained over time, it reached smokers across the province including some groups that may be more likely to experience barriers to accessing treatment, and it successfully helped smokers achieve and maintain abstinence from smoking. A high rate of adoption was largely maintained over time, demonstrating the continued demand for this service in the province. This was evident despite the fact that during this time the STOP program had also expanded its reach through partnerships with other health care organizations across the province, which by 2016 had included 237 primary care organizations and 54 addictions agencies. A greater proportion of PHUs adopted STOP on the Road compared to the pilot program, suggesting the workshops were easier for PHUs to implement than brief individual counselling. Furthermore, a high level of adoption by PHUs was recovered after a temporary cessation of workshops due to lack of funding; therefore, this model may be well suited to other settings where funding may not be stable. Each PHU is autonomous, governed by its own board of health, with its own unique mandate and strategic priorities that may vary over time. Thus, one organizational barrier to adoption was that workshops may not have been aligned with an individual PHU’s current priorities. For example, one PHU no longer provides direct services to individuals and therefore does not offer workshops.

The proportion of eligible smokers reached was somewhat higher compared to Ontario’s quitline [44, 45]. Reach was similar to reports for other free NRT giveaway programs that provided up to 2 weeks of free NRT, though it was lower than the reach of one program that provided 6 weeks [46]. Similar to these previous giveaway programs, reach was limited by the amount of NRT available for distribution. Additional funding may have enabled a greater number of smokers to be reached. Every PHU in Ontario at some point offered a workshop during the 10-year period, thus the program was able to reach communities across all of Ontario, including rural communities which are more likely to be underserved. According to the Canadian census, during the evaluation timeframe approximately 14–15% of the population in Ontario resided in a rural area (according to their definition) [47, 48]. To the extent that the definitions of rural area are comparable, this suggests there was equitable reach for rural residents in most years. However, the proportion of rural residents unexpectedly decreased in 2015–2016 and needs additional monitoring and investigation to ensure there is continued reach in rural communities. The program was also more likely to reach particular subgroups of smokers that may face increased difficulty with quitting and greater barriers to treatment, such as those with lower income [49] or with a mood disorder diagnosis [50, 51]. Approximately one-third of our sample reported an annual household income before tax of less than $20,000, which for most households is below the threshold the Government of Canada sets to classify households as having a low income [52]. This figure is considerably higher than the prevalence of low-income in Ontario around this time (11% in 2005 and 10% in 2015) [53]. However, as we did not have data available on barriers to accessing treatment, we can only speculate on how this program may have improved access to treatment for particular groups, such as those with low income (i.e., annual household income <$20,000) or those living in rural communities.

The 7-day point prevalence abstinence rates we report are similar to those of various free NRT giveaway programs [46]. While year-to-year comparisons are problematic due to varying response rates, effectiveness did not appear to decrease once PHUs began delivering the workshops independently, as expected given the primary active component of the intervention was the NRT and the material delivered was standardized.

This program evaluation demonstrates that an outreach program using workshops to deliver smoking cessation treatment is effective and easily adoptable by existing public health organizations. There were only opportunity costs for the implementer, which was the time devoted to implementation and training. However, this was an appropriate use of their resources and allowed PHUs to meet their standard of addressing smoking cessation in their community. However, the program as we implemented it does require some pre-existing infrastructure that may not be present in some jurisdictions. Initially a small, centralized team was responsible for coordinating and travelling to deliver the intervention in various communities, with the public health staff role primarily being recruitment from the local community. This particular model may be more easily extended to other jurisdictions without this level of pre-existing infrastructure, as alternative partners or methods may be used to engage with the local community for the purpose of recruitment. Capacity building so that each site can deliver the intervention independently is not required in order to implement this program, but we have demonstrated how it can permit workshops to be offered more regularly without incurring increased travel expenses.

This program can be implemented to improve access to treatment in settings where there are insufficient resources for establishing a quitline or smoking cessation clinic. Quitlines that provide free medication have higher reach but require greater infrastructure and resources to set up and operate. Since Ontario’s quitline does not provide free pharmacotherapy, we were unable to compare the treatment and cost-effectiveness of providing NRT in workshops versus through a quitline. Workshops initially were offered on a more infrequent basis and individuals waiting for treatment may have dropped out due to waning motivation. In this manner, quitlines are more effective in reaching people when they are ready and motivated to quit. However, offering workshops more frequently mitigates this problem. The workshops’ group format allows more people to be served with limited resources. In addition, the social modeling of quitting behaviour may be advantageous in smaller communities where groups of people quit together. This has been demonstrated in the community dynamics of smoking behaviour in the town of Framingham [54]. While it is encouraging to see that the program is able to reach smokers that may have greater barriers to accessing treatment (i.e., low income, rural residence) or to achieving long-term abstinence (e.g., psychiatric comorbidity), they may require additional or more intensive treatment in order to achieve long-term success. Though we provided participants with a list of resources for seeking additional cessation support, additional free NRT was not available through these programs. There was also no opportunity for prescription medication such as varenicline or bupropion, since no prescriber was involved in the process.

A few limitations of the evaluation are noted. We were not able to evaluate the intervention fully on all dimensions of the RE-AIM framework. We did not have a measure of Implementation at the organizational level; assessment of time and costs was challenging since they were shared and not formally tracked. We also did not monitor or measure fidelity to the protocol, though we took measures to prevent or discourage modification of the intervention components. However, it is possible some adaptation to the local setting would have in fact been beneficial and could be explored in a future study. Response to the follow-up survey was not high and was especially low once we switched to the more cost-effective method of conducting follow-ups exclusively via online survey. With insufficient resources to supplement with telephone surveys, those without Internet access or sufficient digital literacy may have been unable to respond to follow-up and are not represented in the outcome data from more recent years. Thus, our evaluation of effectiveness may have been biased due to nonresponse and proportion quit may have been over (or under) estimated. Finally, our evaluation employed only quantitative methods and a mixed methods approach could have enriched our understanding and appraisal of the intervention on each dimension [55].

## Conclusions

To our knowledge, this is the first report to describe the use of implementation science models and evaluation for public health impact of an outreach program to provide evidence-based smoking cessation treatment to smokers wanting to quit. This model can be adopted in other jurisdictions where there is a gap in smoking cessation services. Additional research is needed to identify and address the limitations of this program and assess the cost-effectiveness and compare it with other programs.

## Abbreviations

CCHS: Canadian Community Health Survey
NRT: Nicotine replacement therapy
PHU: Public health unit
RIO: Rurality Index of Ontario
STOP: Smoking Treatment for Ontario Patients
TEACH: Training Enhancement in Applied Cessation Counselling and Health
